# Prediction of mortality in adult patients with sepsis using six biomarkers: a systematic review and meta-analysis

**DOI:** 10.1186/s13613-019-0600-1

**Published:** 2019-11-08

**Authors:** Andreas Pregernig, Mattia Müller, Ulrike Held, Beatrice Beck-Schimmer

**Affiliations:** 1 Institute of Anesthesiology, University of Zurich, University Hospital Zurich, Rämistrasse 100, CH-8091 Zurich, Switzerland; 2 Department of Biostatistics, Epidemiology, Biostatistics and Prevention Institute, University of Zurich, Hirschengraben 84, 8001 Zurich, Switzerland

**Keywords:** Sepsis, Biomarker, Prognosis, Mortality, Systematic review, Meta-analysis

## Abstract

**Background:**

Angiopoietin-1 (Ang-1) and 2 (Ang-2), high mobility group box 1 (HMGB1), soluble receptor for advanced glycation endproducts (sRAGE), soluble triggering receptor expressed on myeloid cells 1 (sTREM1), and soluble urokinase-type plasminogen activator receptor (suPAR) have shown promising results for predicting all-cause mortality in critical care patients. The aim of our systematic review and meta-analysis was to assess the prognostic value of these biomarkers for mortality in adult patients with sepsis.

**Methods:**

A systematic literature search of the MEDLINE, PubMed, EMBASE, and Cochrane Library databases, for articles in English published from 01.01.1990 onwards, was conducted. The systematic review focused exclusively on observational studies of adult patients with sepsis, any randomized trials were excluded. For the meta-analysis, only studies which provide biomarker concentrations within 24 h of admission in sepsis survivors and nonsurvivors were included. Results are presented as pooled mean differences (MD) between nonsurvivors and survivors with 95% confidence interval for each of the six biomarkers. Studies not included in the quantitative analysis were narratively summarized. The risk of bias was assessed in all included studies using the Quality in Prognosis Studies (QUIPS) tool.

**Results:**

The systematic literature search retrieved 2285 articles. In total, we included 44 studies in the qualitative analysis, of which 28 were included in the meta-analysis. The pooled mean differences in biomarker concentration (nonsurvivors − survivors), measured at onset of sepsis, are listed as follows: (1) Ang-1: − 2.9 ng/ml (95% CI − 4.1 to − 1.7, *p* < 0.01); (2) Ang-2: 4.9 ng/ml (95% CI 2.6 to 7.1, *p* < 0.01); (3) HMGB1: 1.2 ng/ml (95% CI 0.0 to 2.4, *p* = 0.05); (4) sRAGE: 1003 pg/ml (95% CI 628 to 1377, *p* < 0.01); (5) sTREM-1: 87 pg/ml (95% CI 2 to 171, *p* = 0.04); (6) suPAR: 5.2 ng/ml (95% CI 4.5 to 6.0, *p* < 0.01).

**Conclusions:**

Ang-1, Ang-2, and suPAR provide beneficial prognostic information about mortality in adult patients with sepsis. The further development of standardized assays and the assessment of their performance when included in panels with other biomarkers may be recommended.

*Trial registration* This study was recorded on PROSPERO, prospective register of systematic reviews, under the registration ID: CRD42018081226

## Background

The burden of sepsis stays considerably high to this day [1]. Estimates suggest it affects millions of people worldwide and annually causes nearly 6 million deaths [2]. Due to its aggressive course, it requires rapid recognition and urgent treatment. One challenge remains to accurately identify patients with a higher risk of mortality, and who might benefit from additional monitoring or treatment measures. As sepsis is a highly intricate condition and its clinical assessment is often difficult, the additional use of biomarkers to help pinpoint such patients is an attractive solution. But the heterogeneity and complex pathophysiology of sepsis entail that single biomarkers often provide imprecise information, and biomarkers which reliably qualify as predictors of outcome in sepsis patients remain scarce [3–5].

Recent years have seen the emergence of promising biomarkers. These include angiopoietin 1 (Ang-1) and 2 (Ang-2), high mobility group box 1 protein (HMGB1), soluble receptor for advanced glycation endproducts (sRAGE), soluble triggering receptor expressed on myeloid cells 1 (sTREM1), and soluble urokinase-type plasminogen activator receptor (suPAR). Other promising biomarkers exist, but they do not predict the outcome of sepsis. Moreover, analysis of some biomarkers is extremely complex, which could hamper their future implementation into a clinical setting.

Angiopoietins 1 and 2 are glycoproteins which act on angiogenesis and have a direct, but opposing, effect on blood vessels. Ang-1 supports stabilization, survival and development of endothelial cells, and has anti-inflammatory properties while Ang-2 is proinflammatory, induces endothelial cell destabilization and vascular leakage, and promotes cell death [6]. Therefore, for prediction purposes, both proteins have to be evaluated at the same time.

HMGB1 is a nuclear protein with proinflammatory features when released by dying cells or secreted by immune and inflammatory cells such as neutrophils, monocytes and macrophages in response to infectious or non-infectious stimuli [7].

The cell-surface receptors RAGE, TREM-1, and uPAR are found and measured in the blood as their soluble form: sRAGE, sTREM-1, and suPAR, respectively. RAGE is a cell-surface receptor of the immunoglobulin superfamily, primarily expressed in the lung. When stimulated, RAGE leads to cell activation and the initiation and propagation of an inflammatory response [8]. TREM-1 is found on neutrophils and monocytes. Infection stimulates the expression of the receptor, and its activation leads to a heightened production of inflammatory cytokines [9, 10]. Finally, uPAR is present on immune cells including monocytes and T-lymphocytes, as well as non-immune cells such as endothelial cells and fibroblasts. This marker is notably implicated in cell adhesion, chemotaxis, immune activation and cellular signaling [11].

Clinical studies evaluating the performance of these biomarkers to predict mortality in critically ill patients have shown relevant findings [12, 13]. Some have been reviewed by combining evidence from cohorts of patients with trauma, sterile inflammation or bacteremia in addition to patients with overt sepsis or septic shock [14–17]. Confirming the prognostic value of any of these six biomarkers in patients with sepsis could lead to more efficient surveillance or triage in the intensive care unit (ICU) aiming to reduce mortality.

The aim of our systematic review and meta-analysis is therefore to assess the prognostic value at onset of sepsis of serum Ang-1, Ang-2, HMGB1, sRAGE, sTREM-1, and suPAR, in adult patients with sepsis or septic shock.

## Methods

This study was conducted following the Preferred Reporting Items for Systematic Reviews and Meta-analyses (PRISMA) guidelines [18]. It was recorded on PROSPERO, the prospective register of systematic reviews, under the registration ID: CRD42018081226.

### Search strategy

An information specialist conducted a systematic literature search of the MEDLINE, PubMed, EMBASE, and Cochrane Library databases, for articles in English, published from 01.01.1990 onwards and conducted on humans. The full search strategy is detailed in the Additional file 1. We additionally screened the reference lists of selected studies and of related systematic reviews, to identify any relevant studies not found by the electronic search.

### Study selection

Studies were first screened by title and/or by abstract. For studies included after title/abstract screening, full texts were obtained for formal inclusion or exclusion into our study. Studies were selected independently by two review authors (AP, MM). Discrepancies were resolved by consensus or by arbitration with a third author if necessary (BBS).

### Inclusion and exclusion criteria

This study includes only observational studies which provide prognostic information on one of the biomarkers in adult (≥ 16 years) patients with sepsis, and which applied either the sepsis-1, sepsis-2, and/or sepsis-3 definitions [19]. Prognostic information was defined as all-cause mortality, at any timepoint. Reviews, letters, commentaries, correspondences, case reports, conference abstracts, expert opinions, editorials, in vitro and animal experiments and interventional studies (randomized or non-randomized) were excluded to allow evaluation of similarly retrieved data.

In case of publications with overlapping cohorts or duplicate data, only the publication with the highest number of patients was included.

### Data extraction and analysis

The following data were extracted from published articles and supplementary material if available: (i) general study information: author, year, country, study design (prospective or retrospective), clinical setting; (ii) patient characteristics: sample size, age, male proportion, severity of sepsis, sepsis definition used; (iii) biomarker measurement: time point of measurement, assay; (iv) mortality: follow-up duration, rate of mortality (v) outcome measures: biomarker concentration in survivors and nonsurvivors, area under the receiver operating characteristic (ROC) curve for prediction of mortality with cut-off point, sensitivity, specificity, positive predictive value, negative predictive value, positive likelihood ratio, and negative likelihood ratio.

The data were recorded independently and in duplicate by two review authors (AP, MM) on separate copies of an excel spreadsheet. These were compared, and any discrepancies were resolved by consensus. Data not directly available in published articles was provided by some study authors directly, or calculated based on datasets provided by authors.

Quality assessment was conducted using the Quality in Prognosis Studies (QUIPS) tool [20]. Each study was assessed for risk of bias through six domains: study participation, study attrition, prognostic factor measurement, confounding measurement and account, outcome measurement, analysis and reporting. For each domain, two review authors (AP, MM) independently assigned a rating of low, moderate, or high risk of bias. Again, discrepancies were resolved through discussion.

To assess the prognostic value of the biomarkers for mortality, a meta-analysis of differences in biomarker levels between nonsurvivors and survivors of sepsis was performed, and the results of ROC analyses within studies for prediction of mortality according to biomarker levels were narratively summarized.

The meta-analysis includes studies which provide biomarker concentrations at baseline (within 24 h of admission) in sepsis survivors and nonsurvivors. Studies which did not provide biomarker concentrations at baseline (within 24 h of admission) in sepsis survivors and nonsurvivors were not included in the quantitative analysis, and were narratively summarized.

For pooling of the results, reported means with standard deviations (SD) were used for calculations. If studies reported means with standard errors (SE), the SD was computed using the formula provided by the Cochrane Collaboration: SD = SE * √*N* [21]. For studies which reported biomarker concentrations as median and range or interquartile range (IQR), we estimated the mean and SD according to the formulas by Wan et al. [22]. To confirm the reliability of these estimations, we performed them in duplicate using the formulas by Luo et al. [23], and conducted a sensitivity analysis to compare the results of the two methods. Both methods have shown good reliability for these estimations, even in presence of deviation from the normal distribution [24].

Results are presented as forest plots of pooled mean differences (MD) between nonsurvivors and survivors with 95% confidence interval, separately for each of the six biomarkers. Statistical significance was defined at the 5% level (*p* < 0.05). Heterogeneity was measured using among-study variance (*τ*^2^), the *χ*^2^ test, and the *I*^2^ statistic. For biomarkers with an *I*^2^ < 50%, results were pooled using a fixed effects model, otherwise a random effects model was used. All statistical analyses were performed using R (version 3.4.3) [25], and using the dplyr [26], ggplot2 [27], and meta [28] packages.

## Results

### Study selection and characteristics

**Fig. 1:**
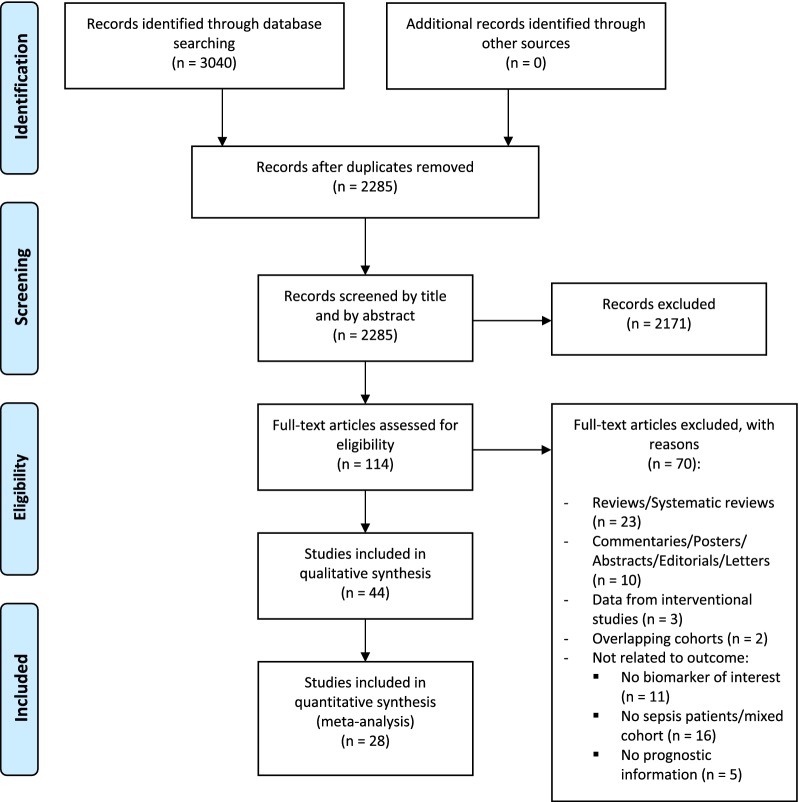
PRISMA flow diagram of study selection

The systematic literature search retrieved 2285 articles (Fig. 1). After initial screening by title and/or by abstract, 2171 articles were excluded. The full texts of the remaining 114 articles were examined, and 70 studies were further excluded. A list of the excluded articles with reasons is available (Additional file 2). In total, we included 44 studies in the qualitative analysis, of which 28 were included in the meta-analysis. All studies included in the qualitative analysis were published between 2005 and 2017. Forty-one of the 44 studies followed a prospective design, whereas 3 were retrospective. All of the included studies sampled blood at admission or enrollment in the study for biomarker determination. The biomarkers were widely measured using commercials assays, but in 2 studies (both for HMGB1) non-commercial methods were used for the measurements.

The main outcome was either 28-day mortality, 30-day mortality, ICU mortality, in hospital mortality, or 90-day mortality. Details regarding the study design and population, assay used, and mortality follow-up of included studies are presented in Table 1.

**Table 1:** Characteristics of all included studies

Author	Year	Country	Study design	*N*	Assay	Outcome
Angiopoietin 1 and 2						
Parikh [35]	2006	USA	Prospective	22	ELISA (R&D Systems)	Hospital mortality
Kranidioti [31]	2009	Greece	Prospective	90	ELISA (R&D Systems)	28-day mortality
Siner [37]	2009	USA	Prospective	66	ELISA (R&D Systems)	28-day, ICU, and hospital mortality
van der Heijden [38]	2009	Netherlands	Prospective	50	ELISA (R&D Systems)	28-day, ICU, and hospital mortality
Davis [29]	2010	Australia	Prospective	124	ELISA (R&D Systems)	28-day mortality
Ricciuto [36]	2011	Canada	Retrospective	70	ELISA (R&D Systems)	28-day mortality
Fang [30]	2015	China	Prospective	495	ELISA (Abcam)	28-day mortality
Lin [32]	2015	Taiwan	Prospective	96	ELISA (Sekisui Diagnostics)	Hospital mortality
Mikacenic [33]	2015	USA	Retrospective	943	Multiplex immunoassay (Meso Scale Discovery)	28-day mortality
Palud [34]	2015	France	Prospective	20	ELISA (RayBiotech)	7-day, 10-day, and 28-day mortality
Total number of patients = 1976						
HMGB1						
Sunden-Cullberg [39]	2005	Sweden	Prospective	64	Western immunoblotting (Cocalico Biologicals)	28-day mortality
Gibot [40]	2007	France	Prospective	42	ELISA (Shino-Test Corporation, customized)	28-day mortality
van Zoelen [41]	2007	Belgium	Prospective	111	ELISA (non-commercial)	Hospital mortality
Karlsson [42]	2008	Finland	Prospective	257	ELISA (Shino-Test Corporation)	ICU and hospital mortality
Huang [43]	2011	China	Prospective	131	ELISA (Shino-Test Corporation)	Not specified
Ueno [44]	2011	Japan	Prospective	60	ELISA (non-commercial)	Not specified
Narvaez-Rivera [45]	2012	Mexico	Prospective	30	ELISA (IBL International)	28-day mortality
Charoensup [46]	2014	Thailand	Prospective	77	ELISA (IBL International)	1-month mortality
Ravetti [47]	2015	Brazil	Prospective	75	ELISA (IBL International)	28-day and ICU mortality
Lee [48]	2016	Korea	Prospective	212	ELISA (IBL International)	28-day, ICU, and Hospital mortality
Nobre [49]	2016	Brazil	Prospective	62	ELISA (IBL International)	28-day and ICU mortality
Total number of patients = 1121						
sRAGE						
Bopp [50]	2008	Germany	Prospective	37	ELISA (R&D Systems)	28-day mortality
Narvaez-Rivera [45]	2012	Mexico	Prospective	30	ELISA (R&D Systems)	28-day mortality
Brodska [51]	2013	Czech Republic	Prospective	54	ELISA (R&D Systems)	28-day mortality
Hamasaki [52]	2014	Brazil	Prospective	73	Multiplex immunoassay (EMD Millipore)	Not specified
Total number of patients = 194						
sTREM-1						
Gibot [53]	2005	France	Prospective	63	Immunoblotting (R&D Systems)	28-day mortality
Giamarellos-Bourboulis [54]	2006	Greece	Prospective	90	ELISA (R&D Systems, customized)	28-day mortality
Phua [55]	2008	Singapore	Prospective	93	Immunoblotting (R&D Systems)	28-day mortality
Suarez-Santamaria [56]	2010	Spain	Prospective	253	ELISA (R&D Systems, customized)	7-day, 28-day, hospital, 6-months, and 1-year mortality
Zhang [57]	2011	China	Prospective	52	ELISA (R&D Systems)	28-day mortality
Su [58]	2012	China	Prospective	160	ELISA (R&D Systems)	28-day mortality
Li [59]	2014	China	Prospective	102	ELISA (R&D Systems)	28-day mortality
Bayram [60]	2015	Turkey	Prospective	74	ELISA (R&D Systems)	Not specified
Ravetti [47]	2015	Brazil	Prospective	75	ELISA (R&D Systems)	28-day and ICU mortality
Charles [61]	2016	France	Prospective	190	ELISA (R&D Systems)	14-day and ICU mortality
Brenner [62]	2017	Germany	Retrospective	120	ELISA (R&D Systems)	90-day mortality
Total number of patients = 1272						
suPAR						
Giamarellos-Bourboulis [63]	2012	Greece	Prospective	1914	ELISA (ViroGates)	28-day mortality
Gustafsson [64]	2012	Sweden	Prospective	49	ELISA (ViroGates)	90-day mortality
Hoenigl [65]	2013	Austria	Prospective	132	ELISA (ViroGates)	28-day mortality
Suberviola [66]	2013	Spain	Prospective	137	ELISA (ViroGates)	ICU and hospital mortality
Donadello [67]	2014	Belgium	Prospective	258	ELISA (ViroGates)	28-day and ICU mortality
Khater [68]	2016	Egypt	Prospective	80	ELISA (R&D Systems)	30-day mortality
Liu [69]	2016	China	Prospective	137	ELISA (ViroGates)	28-day mortality
Shan [70]	2016	China	Prospective	142	ELISA (commercial, not specified)	90-day mortality
Tsirigotis [71]	2016	Greece	Prospective	105	ELISA (ViroGates)	28-day and ICU mortality
Zeng [72]	2016	China	Prospective	126	ELISA (USCN Life Science)	28-day mortality
Total number of patients = 3080						

*ELISA* enzyme-linked immunosorbent assay, *ICU* intensive care unit

### Angiopoietin 1 and 2

Ten of the 44 included studies involve Ang-1 and Ang-2 [29–38]. The number of patients across these studies ranged from 20 to 943, and the mean or median age of the patients from 51 to 75 years.

**Fig. 2:**
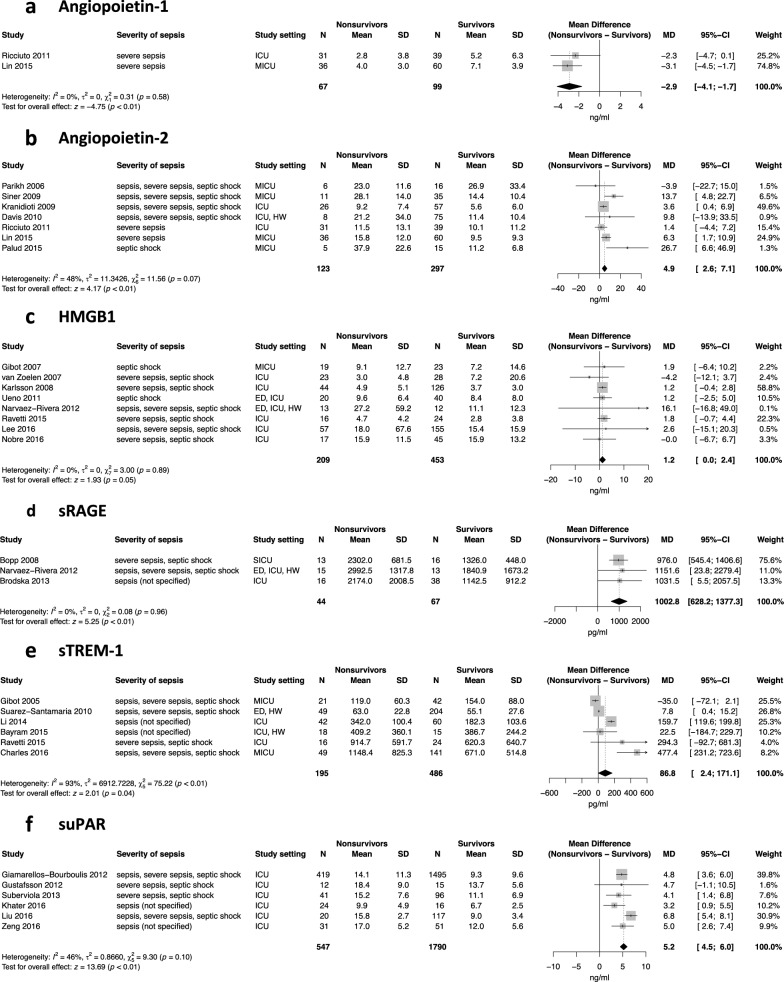
Forest plots of pooled mean differences in biomarker concentration (nonsurvivors − survivors). Effect estimates to the left of 0 indicate higher biomarker concentrations in survivors. Effect estimates to the right of 0 indicate higher biomarker concentrations in nonsurvivors. *SD* standard deviation, *MD* mean difference; setting of study: *ED* emergency department, *ICU* intensive care unit, *MICU* medical intensive care unit, *SICU* surgical intensive care unit, *HW* hospital ward

For the meta-analysis, 2 studies had suitable data for Ang-1 and 7 studies for Ang-2. There is strong evidence that both Ang-1 and Ang-2 measured at onset of sepsis differ between nonsurvivors and survivors. Ang-1, the only anti-inflammatory biomarker included in this review, was lower in nonsurvivors than in survivors, with a pooled mean difference of − 2.9 ng/ml (95% CI − 4.1 to − 1.7; *p* < 0.01). On the contrary, Ang-2 was higher in nonsurvivors than in survivors. The pooled mean difference for Ang-2 is 4.9 ng/ml (95% CI 2.6 to 7.1; *p* < 0.01). All results of the meta-analysis are presented in the forest plots of Fig. 2.

Heterogeneity between studies was not considered substantial in the analyses of Ang-1 and Ang-2, with I^2^ values of 0% for Ang-1 (*p* = 0.58) and 48% for Ang-2 (*p* = 0.07). Therefore, a fixed effects model was used for the analyses.

To further determine the prognostic value of the biomarkers, results of receiver operating characteristic (ROC) analyses for prediction of mortality according to day 1 biomarker concentrations were summarized. Six of the 10 studies reported data for ROC analyses of Ang-1 and/or Ang-2. The area under the curve (AUC) ranged from 0.620 to 0.778 for Ang-1 and 0.632 to 0.960 for Ang-2. The results with details regarding optimal cutoff points and sensitivity, specificity, positive predictive value (PPV), negative predictive value (NPV), positive likelihood ratio (LR+) and negative likelihood ratio (NPV) are presented in Table 2.

**Table 2:** ROC analyses for prediction of mortality according to baseline (< 24 h of admission) biomarker concentration

Study	Mortality follow-up	AUC (95% CI)	Cutoff	Sn	Sp	PPV	NPV	LR+	LR−
Angiopoietins 1 and 2									
Ang-2/Ang-1 ratio									
Fang [30]	28 days	0.845 (0.810 to 0.880)	1.94	80%	81%	78%	83%	3.98	0.31
Ang-1									
Ricciuto [36]	28 days	0.620 (0.500 to 0.760)	–	–	–	–	–	–	–
Lin [32]	During hospital stay	0.743 (0.726 to 0.847)	–	–	–	–	–	–	–
Fang [30]	28 days	0.778 (0.732 to 0.824)	–	–	–	–	–	–	–
Ang-2									
Lin [32]	During hospital stay	0.632 (0.515 to 0.750)	–	–	–	–	–	–	–
Kranidioti [31]	28 days	0.703 (0.578 to 0.827)	9700 pg/ml	42%	82%	51%	76%	2.32	0.49
van der Heijden [38]	ICU mortality	0.790	3066 pg/ml	73%	71%	39%	91%	2.52	0.38
Fang [30]	28 days	0.794 (0.750 to 0.837)	–	–	–	–	–	–	–
Palud [34]	28 days	0.960 (0.870 to 1.050)	26,780 pg/ml	100%	93%	83%	100%	14.29	0
HMGB1									
Karlsson [42]	During hospital stay	0.570 (0.470 to 0.670)	6.5 ng/ml	39%	79%	40%	79%	1.87	0.77
Gibot [40]	28 days	0.610	–	–	–	–	–	–	–
sRAGE									
Bopp [50]	28 days	–	1569 pg/ml	85%	75%	73%	86%	3.38	0.21
Brodska [51]	28 days	0.660 (0.492 to 0.827)	1804 pg/ml	63%	76%	53%	83%	2.64	0.49
sTREM-1									
Bayram [60]	not specified	0.444	255 pg/ml	50%	40%	50%	40%	0.83	1.25
Suarez-Santamaria [56]	28 days	0.598 (0.520 to 0.676)	55.7 pg/ml	68%	58%	31%	87%	1.62	0.55
Charles [61]	ICU mortality	0.640 (0.540 to 0.740)	954 pg/ml	55%	78%	49%	82%	2.48	0.58
Ravetti [47]	28 days	0.640 (0.460 to 0.830)	750 pg/ml	56%	68%	60%	65%	1.75	0.65
Ravetti [47]	ICU mortality	0.690 (0.510 to 0.870)	750 pg/ml	56%	75%	60%	72%	2.24	0.59
Gibot [53]	28 days	0.740 (0.680 to 0.800)	180 pg/ml	86%	70%	59%	91%	2.87	0.20
Su [58]	28 days	0.748 (0.637 to 0.860)	0.499	63%	84%	81%	54%	4.01	0.43
Brenner [62]	90 days	0.827	521 pg/ml	71%	86%	87%	79%	4.99	0.33
Li [59]	28 days	0.856 (0.784 to 0.929)	252 pg/ml	86%	76%	71%	88%	3.53	0.19
suPAR									
Suberviola [66]	During hospital stay	0.670 (0.570 to 0.770)	9.6 ng/ml	81%	46%	39%	85%	1.49	0.43
Giamarellos-Bourboulis [63]	28 days	0.708 (0.681 to 0.736)	12.0 ng/ml	62%	69%	36%	87%	1.99	0.55
Khater [68]	30 days	0.720 (0.560 to 0.853)	6.3 ng/ml	79%	63%	76%	67%	2.11	0.33
Donadello [67]	ICU and 28 days	0.723	10.2 ng/ml	71%	65%	38%	88%	2.03	0.45
Zeng [72]	28 days	0.765 (0.658 to 0.872)	12.0 ng/ml	87%	73%	66%	90%	3.17	0.18
Shan [70]	90 days	0.780 (0.630 to 0.930)	310 pg/ml	69%	65%	–	–	–	–
Tsirigotis [71]	28 days	0.787	7.6 ng/ml	82%	73%	63%	88%	3.04	0.25
Liu [69]	28 days	0.788 (0.73 to 0.846)	10.8 ng/ml	85%	78%	39%	97%	3.79	0.19

*ICU* intensive care unit, *ROC* receiver operating characteristic, *AUC* area under the curve, *Sn* sensitivity, *Sp* specificity, *PPV* positive predictive value, *NPV* negative predictive value, *LR*+ positive likelihood ratio, *LR*− negative likelihood ratio

One study [33] did not report biomarker concentrations or ROC analyses, but still contains prognostic information. This study included patients with systemic inflammatory response syndrome (SIRS), a subset of whom had sepsis. The authors retrospectively performed a multivariate analysis of biomarker association with 28-day mortality in the sepsis patients. After adjustment for various variables (including age, gender and comorbidities), they report odds ratios (OR) for mortality at 28 days of 0.69 (95% CI 0.59 to 0.81; *p* = 2.9 × 10^−6^) per doubling of Ang-1 and 1.79 (95% CI 1.43 to 2.24; *p* = 2.9 × 10^−6^) per doubling of Ang-2.

### HMGB1

Out of 44 included studies 11 discuss HMGB1 [39–49]. The sample size of the studies varied between 30 and 257 patients. The patients were aged 34 (mean or median) to 68 years. The reason for the low age of patients, is because the study by Huang et al. [43] included patients with sepsis in burn patients.

Eight of those studies had suitable data for the meta-analysis. HMGB1 measured at onset of sepsis is the only biomarker that did not differ between nonsurvivors and survivors of sepsis, with a pooled mean difference of 1.2 ng/ml (95% CI 0.0 to 2.4; *p* = 0.05) (Fig. 2).

There was no significant heterogeneity between studies for HMGB1 (*I*^2^ = 0%; *p* = 0.89), so a fixed effects model was used for the pooling of the results.

Only 2 of the 11 studies contained ROC analyses, with AUCs of 0.570 and 0.610, respectively (Table 2).

In addition, 3 studies did not report sufficient data to be included in the meta-analysis and did not perform ROC analyses [39, 43, 46].

Sunden-Cullberg et al. studied the kinetics of HMGB1 in sepsis patients. They measured HMGB1 in sepsis nonsurvivors and survivors using two different (non-commercial) methods. They report conflicting results, one method showing lower HMGB1 levels in nonsurvivors than in survivors, the other method showing no difference between the two groups.

The study by Huang et al. includes burn patients, some of which developed sepsis. In this subgroup of sepsis patients, they reported no significant difference in HMGB1 concentrations on postburn day 1 between nonsurvivors and survivors.

Charoensup et al. studied a specific cohort of patients with sepsis due to *Burkholderia pseudomallei*, and they report that nonsurvivors had higher HMGB1 levels than survivors at the time of diagnosis.

### sRAGE

For sRAGE only 4 studies could be included [45, 50–52]. The sample size of these studies ranged from 30 to 73 patients. The patients had a mean or median age of 38 to 64 years.

Three of the 4 studies were suitable for the meta-analysis. Our results show strong evidence that sRAGE is higher in nonsurvivors than survivors. The pooled mean difference for sRAGE is 1003 pg/ml (95% CI 628 to 1377; *p* < 0.01) (Fig. 2).

There was no substantial heterogeneity between studies, with an *I*^2^ value of 0% (*p* = 0.96), so we used a fixed effects model for pooling.

Two of the studies had data regarding ROC analyses. While optimal cutoff points are summarized for both studies, only one reported an AUC with a value of 0.660 (Table 2).

One study [52] could not be included in the meta-analysis and did not report ROC analysis. Hamasaki et al. indicate that increased levels of sRAGE in septic shock patients are associated with mortality.

### sTREM-1

Eleven of the 44 included studies involve sTREM-1 [47, 53–62]. The sample size of patients in the different studies ranged from 52 to 253 patients. A mean or median age of 53 years to 70 years was identified.

Six of these studies were suitable for meta-analysis. sTREM-1 was significantly higher in nonsurvivors, but to a lesser degree than the other biomarkers. The pooled mean difference for sTREM-1 is 87 pg/ml (95% CI 2 to 171; *p* = 0.04) (Fig. 2).

Heterogeneity between studies was considered significant for the analyses of sTREM-1 with an *I*^2^ of 93% (*p* < 0.01), so a random effects model was used for pooling of the results.

ROC analyses were reported in 8 studies. The AUCs had varying values, from 0.444 to 0.856 (Table 2).

Three studies did not report sufficient data for the meta-analysis or details of ROC analyses [54, 55, 57].

Giamarellos-Bourboulis et al. studied the kinetics of sTREM-1 in patients with sepsis due to ventilator-associated pneumonia (VAP). On day 1, they found significantly higher levels of sTREM-1 in nonsurvivors compared to survivors.

Phua et al. did not find sTREM-1 to be predictive of mortality in a cohort of patients with septic shock. Zhang et al. evaluated sepsis patients and while they showed that nonsurvivors had higher sTREM-1 concentrations than survivors on day 1, the difference was not significant.

### suPAR

suPAR was determined in 10 of the 44 studies [63–72]. The sample size ranged from 49 to 1914 patients. The age of the patients was consistent, ranging from a mean or median of 59 years to 71 years.

We could include 6 of these studies in the meta-analysis. Our results strongly indicate that suPAR is higher in nonsurvivors than in survivors. The pooled mean difference for suPAR is 5.2 ng/ml (95% CI 4.5 to 6.0; *p* < 0.01) (Fig. 2).

The heterogeneity between studies was not significant (*I*^2^ = 46%; *p* = 0.10), and the results were pooled using a fixed effects model.

Eight of the 10 studies contained data regarding ROC analyses. The AUC for suPAR for prediction of mortality ranged from 0.670 to 0.788 (Table 2).

One study [65] was not included in the meta-analysis and did not contain information regarding ROC analysis. Hoenigl et al. studied suPAR concentration in patients with SIRS or sepsis. In their whole cohort, suPAR concentrations were significantly higher in nonsurvivors than in survivors. In the subgroup of patients with sepsis, they describe that patients with increasing suPAR concentrations within day 1 following admission to the emergency department had a higher mortality rate than those who had decreasing concentrations of suPAR.

### Study quality

**Fig. 3:**
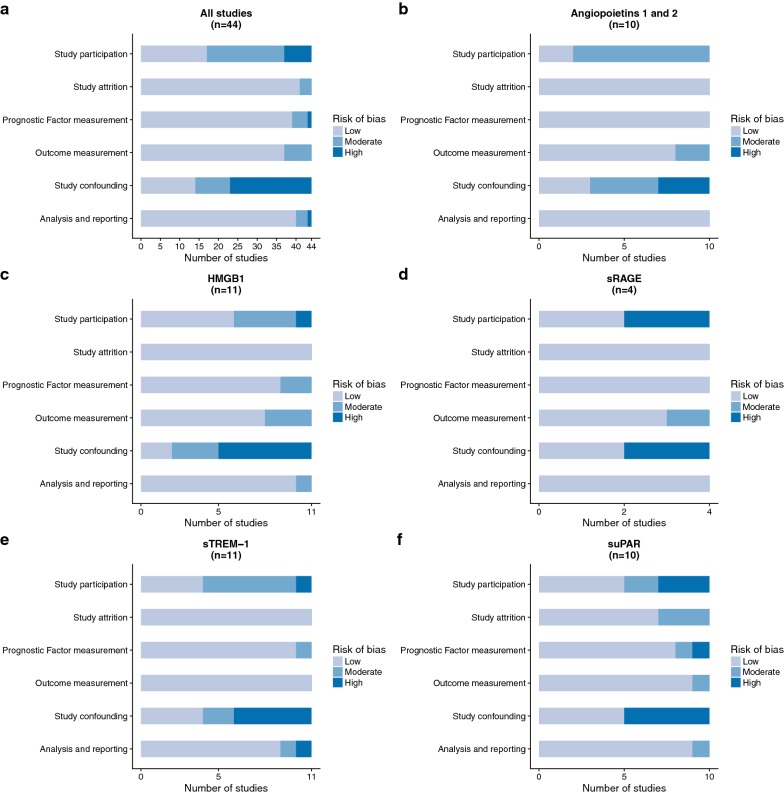
Quality assessment of included studies, according to the six bias domains of the QUIPS tool. **a** Quality of all included studies, **b**–**f** quality of studies for each biomarker

The overall results of quality assessment, as well as the quality of studies for each biomarker, are displayed in Fig. 3. No studies were excluded due to low quality.

We found selection bias to be a concern for more than half of the included studies. This was due to studies not following consecutive recruitment, no or partial definition of inclusion and exclusion criteria as well as time and/or place of recruitment.

Study attrition and risk of attrition bias was very low, as almost no patients were lost to follow-up across studies. The proportion of missing data was also low. The reasons for missing data were often missing blood samples when repeated measurements were made, or, for studies which assessed survival by phone at 90 days, missing information on survival status.

The time and measurement of biomarkers was well described, with only 2 studies not reporting the sampling timeframe.

Mortality was well defined in 40 studies, but 4 studies did not report the follow-up period for mortality.

Confounding was another large source of bias. Only 14 studies adequately recorded confounders (such as comorbidities) and controlled for confounding by restriction or adjustment for the assessment of their outcome. Nine studies partly accounted for confounding. The remaining 21 studies were at high risk of bias due to no or insufficient recognition and control for confounding.

We found 4 studies susceptible to risk of bias due to inadequate statistical analysis or reporting of results. This was engendered by the partial reporting of primary outcomes, insufficient data disclosure, or multiplicity issues due to the reporting of a large number of outcomes, which meant that results were likely to be spurious.

## Discussion

The results of our meta-analysis show that of the 6 biomarkers we evaluated, Ang-1, Ang-2, sRAGE and suPAR are the ones that most highly differ at onset of the disease between patients dying of sepsis and those who survive. sTREM-1 slightly differed between those two groups, but the evidence was less compelling. Our results do not show evidence that HMGB1 distinguishes nonsurvivors from survivors.

The results of ROC analyses further support the predictive value of Ang-1, Ang-2, and suPAR for mortality in sepsis patients. These biomarkers show superior AUC values, specificity and sensitivity than sRAGE, sTREM-1 and HMGB1. Additionally, their AUC values are comparable to those of biomarkers currently clinically in use such as procalcitonin (PCT) [73, 74], or clinical scores such as the Sequential Organ Failure Assessment (SOFA) score [75]. Regarding negative and positive predictive values, in general, all six biomarkers have higher NPVs than PPVs. The PPV between biomarkers is similar, except HMGB1 for which it is lower. All biomarkers show high NPVs, except sTREM-1 which has a lower range of values.

Biomarkers are routinely used in day-to-day clinical practice. In the ICU, few biomarkers other than PCT have demonstrated reliability for the prediction of mortality in sepsis patients, which has prompted the search for new biomarkers [4, 5]. Some of the six biomarkers we evaluated have been analyzed in systematic reviews of studies with varying cohorts of ICU patients. Backes et al. [14] evaluated the diagnostic and prognostic value of suPAR in a narrative review of studies of patients with systemic inflammation, bacteremia, or sepsis. They conclude that suPAR shows encouraging prognostic value, with higher levels being associated with increased mortality. More recently, Ni et al. [17] performed a systematic review with meta-analysis about the diagnostic and prognostic value of suPAR, focusing on patients with bacterial infection with or without sepsis. Their results show that high suPAR is associated with an elevated risk of mortality, with a pooled risk ratio of 3.37 (95% CI 2.60 to 4.38), and an AUC of 0.77 for the prediction of mortality, with pooled sensitivity and specificity of 70% and 72%. Regarding sTREM-1, in another recent systematic review with meta-analysis of patients with infection, Su et al. [16] concluded that it only had moderate prognostic value and is not significant for the prediction of mortality. They reported a pooled risk ratio (RR) of death with elevated sTREM-1 of 2.54 (95% CI 1.77 to 3.65), and an AUC of 0.76 for the prediction of mortality, with pooled sensitivity and specificity of 75% and 66%. Xing et al. [15] reviewed biomarkers of endothelial activation in sepsis, including Ang-1 and Ang-2, and narratively summarized studies which are also included in our review. The recent meta-analysis by Jabaudon et al. [76] supports sRAGE as a predictor of poor outcome in ARDS patients. However, these systematic reviews all had different inclusion criteria and combined cohorts of patients with various diseases.

A strength of our study is the evaluation of multiple biomarkers which intervene in different aspects of sepsis pathophysiology. Additionally, the majority of included studies are prospective, and we selected only studies with cohorts or subgroups of patients with a diagnosis of sepsis according to well defined criteria, not mixed with other ICU patients. Many other biomarkers, which are not yet routinely used clinically emerged in addition to the six biomarkers assessed in this systematic review. However, the measurement methods are complex or expensive, which could slow their future implementation in the clinic, while others are more useful for the diagnosis than for the prognosis of sepsis. This is the reason why we focused on the six biomarkers only. To eliminate any risk of selective reporting, the list of the six biomarkers chosen for this systematic review was determined before the start of the study and published in the PROSPERO protocol (CRD42018081226).

Our study has some limitations. While we had strict inclusion criteria regarding the definition of sepsis, there is still inevitable variability due to the heterogeneous nature of sepsis and the various study populations from different centers. Furthermore, resulting from the lack of standardization, a variety of assays were used for the measuring of the biomarkers. However, even in presence of this heterogeneity the results are consistent across studies which suggests a good robustness of the biomarkers, especially suPAR, Ang-1 and Ang-2.

Mortality was not assessed at the same time point, but the majority of studies assessed mortality at 28 days or during hospital or ICU stay. For the meta-analysis, we used means with SDs of biomarker concentrations to pool the results. As we did not have access to the original data, we had to estimate the mean and SD of biomarker concentrations when they were reported as median and range or IQR. These estimations were performed using the methods by Wan et al. [22], in duplicate according to the methods by Luo et al. [23] to confirm their accuracy and to reduce bias. Both these methods have shown good reliability for these estimations even in presence of deviation from the normal distribution [24]. No difference was found between the estimations using the two methods. In addition, even if bias for the estimation of the mean and SD was present, the bias would be canceled out for the estimation of the mean difference between nonsurvivors and survivors as it would be found in both groups [77]. A lot of studies did not contain data on biomarker concentrations at baseline and could not be included in the meta-analysis. There is a particularly low number of studies for Ang-1 and sRAGE. Statistical pooling of the results of ROC analyses to provide a summary ROC curve for each biomarker was not feasible due to the low number of studies, and more importantly because of the lack of standardization of biomarker measurement methods, which makes the different cutoff points not directly comparable. The results of all ROC analyses reported within studies were therefore narratively summarized.

Some studies performed repeated measurements to assess the dynamics of biomarker concentration over time. Biomarkers which do not clearly distinguish between survivors and nonsurvivors at onset of sepsis (HMGB1 and sTREM-1) could still provide important prognostic information when assessed at a further time point. But as biomarker concentrations over time are less practical to use, and subject to additional variability due to factors such as fluid resuscitation, this was not a focus of our review.

As mentioned previously, the AUC values of the biomarkers assessed, which performed best (Ang-1, Ang-2 and suPAR) are comparable to AUCs reported in the literature for clinical scores or other biomarkers currently used in clinical settings. Within this systematic review, a few of the included studies also reported AUCs for clinical scores. In 3 studies for angiopoietins, the AUCs of Ang-1 and Ang-2 were comparable to those of the Mortality in Emergency Department Sepsis (MEDS), SOFA and Acute Physiology and Chronic Health Evaluation (APACHE) II scores for predicting mortality [30, 32, 36]. In 5 studies for sTREM-1, one study showed a similar AUC between sTREM-1 and APACHE-II, and in the other 4 studies, sTREM-1 had a lower AUC than either the Simplified Acute Physiology Score (SAPS) II, SOFA and/or APACHE-II scores [56, 58–61]. suPAR had worse AUC values than APACHE-II or SOFA for the prediction of mortality in 4 studies, was comparable to SOFA score but worse than APACHE-II in 2 studies, and better than APACHE-II in one study [63, 66–69, 71, 72]. However, as the prognostic value of clinical scores was not a predefined outcome nor an inclusion or exclusion criteria of our systematic review, this comparison was not addressed in a systematic fashion and therefore may be biased. Comparing the value of the six biomarkers to clinical scores (or to other well-known biomarkers), either narratively or quantitatively, deserves to be evaluated as main outcome in future studies.

Additionally, as sepsis is a very heterogeneous condition, it is unlikely that a single biomarker will accurately predict outcome in all cases. Combining multiple biomarkers as a panel might better reflect the individual disease process of each patient, and thus provide added value compared to single biomarkers [12, 78]. But it is still necessary to determine which biomarkers to include in such a panel, and the biomarkers included in this systematic review which performed best (Ang-1, Ang-2, suPAR) are attractive candidates. Further studies evaluating the prognostic value of combinations of multiple biomarkers or biomarkers with clinical scores are encouraged.

## Conclusions

In summary, we evaluated the prognostic value of six different biomarkers at onset of sepsis, by assessing the difference in biomarker concentration between nonsurvivors and survivors, and by reporting their performance for predicting mortality. Of the biomarkers we evaluated, Ang-1, Ang-2, and suPAR provide the most beneficial prognostic information about mortality in adult patients with sepsis. The further development of standardized assays and the assessment of their role when included in panels with other biomarkers may be recommended.

## Supplementary information

**Additional file 1.** Literature search strategy.

**Additional file 2.** List of excluded articles, with reasons.

**Additional file 3.** PRISMA checklist for systematic reviews and meta-analyses.

## Data Availability

All data generated or analyzed during this study are included in this published article.

## Abbreviations

95% CI: 95% confidence interval
Ang-1: angiopoietin-1
Ang-2: angiopoietin-2
APACHE II: Acute Physiology and Chronic Health Evaluation II
ARDS: acute respiratory distress syndrome
AUC: area under the curve
HMGB1: high mobility group box 1
ICU: intensive care unit
IQR: interquartile range
LR+: positive likelihood ratio
LR−: negative likelihood ratio
MD: mean difference
MEDS: Mortality in Emergency Department Sepsis
NPV: negative predictive value
OR: odds ratio
PCT: procalcitonin
PPV: positive predictive value
PRISMA: Preferred Reporting Items for Systematic Reviews and Meta-analyses
QUIPS: Quality in Prognosis Studies
ROC: receiver operating characteristic
RR: risk ratio
SAPS II: Simplified Acute Physiology Score II
SD: standard deviation
SE: standard error
SIRS: systemic inflammatory response syndrome
SOFA: Sequential Organ Failure Assessment
Srage: soluble receptor for advanced glycation endproducts
sTREM1: soluble triggering receptor expressed on myeloid cells 1
suPAR: soluble urokinase-type plasminogen activator receptor
VAP: ventilator-associated pneumonia
