# Supplementary material for: Prediction of mortality in adult patients with sepsis using six biomarkers: a systematic review and meta-analysis
Source: Ann Intensive Care. 2019 Nov 8;9:125. doi: 10.1186/s13613-019-0600-1 (PMC6841861; doi:10.1186/s13613-019-0600-1)
Supplement: Supplementary file 1 — Additional file 1. Literature search strategy. [file 13613_2019_600_MOESM1_ESM.pdf]

Additional file 1 – Literature search strategy

Databases searched:

- MEDLINE
- PubMed
- EMBASE
- Cochrane Library

Wiley Online Library

Cochrane Library

Trusted evidence.
Informed decisions.
Better health.

Log in / Register

To search an exact word(s) use quotation marks, e.g. "hospital" finds hospital; hospital (no quotation marks) finds hospital and hospitals; pay finds paid, pays, paying, payed)

d to t View fewer lines 6

0	Edi 0	#1	sepsis:kw,ti,ab or septic:kw,ti,ab	0	6514
1	Edi 1	#2	biomarker*:kw,ti,ab or "biological marker*":kw,ti,ab or ((interleukin or il) near/1 (4 or 6 or 8 or 10 or "receptor antagonist*" or "blocking agent*")):kw,ti,ab or "advanced glycation endproduct receptor*":kw,ti,ab or "advanced glycation end product receptor*":kw,ti,ab or "receptor* for advanced glycation endproducts":kw,ti,ab or "receptor* for advanced glycation end products":kw,ti,ab or rage:kw,ti,ab or "monocyte chemoattractant protein*":kw,ti,ab or " monocyte chemotactic protein*":kw,ti,ab or "mcp-1":kw,ti,ab or ("high-mobility group" near/1 (b1 or "box 1")):kw,ti,ab or hmgb1:kw,ti,ab or procalcitonin:kw,ti,ab or "c-reactive [illegible]	1	50779
2	Edi 2	#3	mortality:kw,ti,ab or surviv*:kw,ti,ab or "length of stay": kw,ti,ab or "time spent in icu":kw,ti,ab or "time spent in [illegible]	2	139763
3	Edi 3	#4	prognos*:kw,ti,ab or predict*:kw,ti,ab or correlat*:kw,ti,ab or accuracy:kw,ti,ab or accurate:kw,ti,ab or utility:kw,ti,ab or [illegible]	3	157295
4	Edi 4	#5	#1 and #2 and #3 and #4	4	212
6	6	#6		6 6	N/A

ear Strate Search Help

Highlight orphan lines

Save strategy

Strategy Name

Comments

ve Strate

All Results (212)

- Cochrane Reviews (1)
  - All
  - Review
  - Protocol
- Other Reviews (0)
- Trials (211)
- Methods Studies (0)
- Technology Assessments (0)
- Economic Evaluations (0)
- Cochrane Groups (0)

- All
- Current Issue

Me Methodology

Cochrane Central Register of Controlled Trials : Issue 9 of 12, September 2017

There are **211** results from **1087263** records for your search on **#5** - **#1 and #2 and #3 and #4** in **Trials** in the strategy currently being edited

Pages evio | 6 - 1 | 1 - 1 | 6 - 2 | 201 - 211

Sort by Date

Select all | xport a port select

Prospective **evaluation** of a shortened regimen of treatment for acute osteomyelitis and **septic** arthritis in children
Jagodzinski NA , Kanwar R , Graham K and Bache CE
Journal of pediatric orthopedics, 2009, 29(5), 518
Online Publication Date: 2012

Hemofiltrastion does not influence early S-100B serum **levels** in **septic** shock patients receiving stress doses of hydrocortisone or placebo
Mussack T , Briegel J , Schelling G and Jochum M
European journal of medical research, 2005, 10(1), 11
Online Publication Date: 2012

Embase®

Embase Session Results (17 Oct 2017)

No.	Query	Results
#9	#8 NOT ([animals]/lim NOT [humans]/lim) AND [english]/lim NOT [conference abstract]/lim AND [1-1-1990]/sd	1939
#8	#6 AND #7	3615
#7	prognos*:ti OR predict*:ti OR correlat*:ti OR accuracy:ti OR accurate:ti OR utility:ti OR useful*:ti OR value*:ti OR evidence:ti OR assess*:ti OR level*:ti OR determin*:ti OR detect*:ti OR measure*:ti OR marker*:ti OR biomarker*:ti OR evaluat*:ti OR 'prognosis'/de OR 'prognostic factor'/de	3903808
#6	#1 AND #4 AND #5	10546
#5	#2 OR #3	1093927
#4	'mortality'/de OR 'in hospital mortality'/de OR 'survival'/de OR 'length of stay'/de OR mortality:ti OR surviv*:ti OR 'length of stay':ti,ab OR 'time spent in icu':ti,ab OR 'time spent in emergency':ti,ab OR 'on machine ventilation':ti,ab OR ((clinical* NEAR/3 outcome*):ti,ab)	1379780
#3	'sequential organ failure assessment score'/de OR 'sepsis-related organ failure assessment':ti,ab OR 'sequential organ failure assessment':ti,ab OR ((sofa NEAR/3 score*):ti,ab)	6437
#2	'biological marker'/de OR 'interleukin 4'/de OR 'interleukin 6'/de OR 'interleukin 8'/de OR 'interleukin 10'/de OR 'interleukin 1 receptor blocking agent'/de OR 'advanced glycation end product receptor'/de OR 'monocyte chemotactic protein'/de OR 'monocyte chemotactic protein 1'/de OR 'high mobility group b1 protein'/de OR 'procalcitonin'/de OR 'c reactive protein'/de OR 'lactic acid'/de OR 'tumor necrosis factor'/de OR 'soluble triggering receptor expressed on myeloid cells 1'/de OR 'soluble urokinase type plasminogen activator receptor'/de OR 'angiopoietin 1'/de OR 'angiopoietin 2'/de OR biomarker*:ti,ab OR 'biological marker*':ti,ab OR (((interleukin OR il) NEAR/1 (4 OR 6 OR 8 OR 10 OR 'receptor antagonist*' OR 'blocking agent*')):ti,ab) OR 'advanced glycation endproduct receptor*':ti,ab OR 'advanced glycation end product receptor*':ti,ab OR 'receptor* for advanced glycation endproducts':ti,ab OR 'receptor* for advanced glycation end products':ti,ab OR rage:ti,ab OR 'monocyte chemoattractant protein*':ti,ab OR 'monocyte chemotactic protein*':ti,ab OR 'mcp-1':ti,ab OR (('high-mobility group' NEAR/1 (b1 OR 'box 1')):ti,ab) OR hmgb1:ti,ab OR procalcitonin:ti,ab OR 'c-reactive protein':ti,ab OR lactate:ti,ab OR 'lactic acid':ti,ab OR 'tumor necrosis factor alpha':ti,ab OR 'tnf-alpha':ti,ab OR 'tnf-α':ti,ab OR 'soluble triggering receptor expressed on myeloid cells-1':ti,ab OR strem1:ti,ab OR 'soluble urokinase-type plasminogen receptor':ti,ab OR 'soluble urokinase-type plasminogen activator receptor':ti,ab OR supar:ti,ab OR 'soluble upar':ti,ab OR 'angiopoietin 1':ti,ab OR 'angiopoietin 2':ti,ab OR 'ang-1':ti,ab OR 'ang-2':ti,ab	1089482
#1	'sepsis'/exp OR sepsis:ti OR septic:ti	230464

ELSEVIER

© 2017 RELX Intellectual Properties SA. All rights reserved.
Embase, RELX Group and the RE symbol are trade marks of RELX Intellectual Properties SA, used under license.

Ovid®

Wolters Kluwer

My Account | Ask a Librarian | Support & Training | University of Zürich | Help | Logoff

Search | Journals | Books | Multimedia | My Workspace | Mobile

Search History (11)

View Saved

Contract

# ▲	Searches	Results	Type	Actions	Annotations
1	exp Sepsis/ or sepsis.ti. or septic.ti.	129370	Advanced	Display Results | More	
2	Biomarkers/ or interleukin-4/ or interleukin-6/ or interleukin-8/ or interleukin-10/ or interleukin 1 receptor antagonist protein/ or Advanced Glycosylation End Product-Specific Receptor/ or Monocyte Chemoattractant Proteins/ or HMGB1 Protein/ or Calcitonin/ or C-Reactive Protein/ or Lactic Acid/ or Tumor Necrosis Factor-alpha/ or Receptors, Urokinase Plasminogen Activator/ or angiopoietin-1/ or angiopoietin-2/ or biomarker*.ti,ab. or biological marker*.ti,ab. or ((interleukin or il) adj1 ("4" or "6" or "8" or "10" or receptor antagonist* or blocking agent*)).ti,ab. or advanced glycation endproduct receptor*.ti,ab. or advanced glycation end product receptor*.ti,ab. or receptor* for advanced glycation endproducts.ti,ab. or receptor* for advanced glycation end products.ti,ab. or rage.ti,ab. or monocyte chemoattractant protein*.ti,ab. or monocyte chemotactic protein*.ti,ab. or mcp-1.ti,ab. or (high-mobility group adj1 (b1 or box 1)).ti,ab. or hmgb1.ti,ab. or procalcitonin.ti,ab. or c-reactive protein.ti,ab. or lactate.ti,ab. or lactic acid.ti,ab. or tumo?r necrosis factor alpha.ti,ab. or tnf-alpha.ti,ab. or soluble triggering receptor expressed on myeloid cells-1.ti,ab. or strem1.ti,ab. or soluble urokinase-type plasminogen receptor.ti,ab. or soluble urokinase-type plasminogen activator receptor.ti,ab. or supar.ti,ab. or soluble upar.ti,ab. or angiopoietin 1.ti,ab. or angiopoietin 2.ti,ab. or ang-1.ti,ab. or ang-2.ti,ab.	865780	Advanced	Display Results | More	
3	(sepsis-related organ failure assessment or sequential organ failure assessment or (sofa adj3 score*)).ti,ab.	2945	Advanced	Display Results | More	
4	Mortality/ or Hospital Mortality/ or Survival/ or Length of Stay/ or mortality.ti. or surviv*.ti. or length of stay.ti,ab. or time spent in icu.ti,ab. or time spent in emergency.ti,ab. or on machine ventilation.ti,ab. or (clinical* adj3 outcome*).ti,ab.	561715	Advanced	Display Results | More	
5	2 or 3	867750	Advanced	Display Results | More	
6	1 and 4 and 5	2262	Advanced	Display Results | More	
7	(prognos* or predict* or correlat* or accuracy or accurate or utility or useful* or value* or evidence or assess* or level* or determin* or detect* or measure* or marker* or biomarker* or evaluat*).ti. or Prognosis/	3266048	Advanced	Display Results | More	
8	6 and 7	906	Advanced	Display Results | More	
9	8 not (animals not humans).sh.	867	Advanced	Display Results | More	
10	limit 9 to english	819	Advanced	Display Results | More	
11	limit 10 to yr="1990 -Current"	815	Advanced	Display Results | More	

PubMed Advanced Search Builder

YouTube Tutorial

⚠ Quoted phrase not found.

Use the builder below to create your search

Edit Clear

Builder

AND All Fields Show index list

AND All Fields Show index list

or Add to history

History

Download history Clear history

Search	Add to builder	Query	Items found	Time
#9	Add	Search ((((((((sepsis[Title] OR septic[Title]))) AND (((((biomarker*[Title/Abstract] OR "biological marker*"[Title/Abstract] OR (((interleukin[Title/Abstract] OR il)[Title/Abstract] AND (4[Title/Abstract] OR 6[Title/Abstract] OR 8[Title/Abstract] OR 10[Title/Abstract] OR "receptor antagonist*"[Title/Abstract] OR "blocking agent*"[Title/Abstract]))) OR "advanced glycation endproduct receptor*"[Title/Abstract] OR "advanced glycation end product receptor*"[Title/Abstract] OR "receptor* for advanced glycation endproducts"[Title/Abstract] OR "receptor* for advanced glycation end products"[Title/Abstract] OR rage[Title/Abstract] OR "monocyte chemoattractant protein*"[Title/Abstract] OR "monocyte chemotactic protein*"[Title/Abstract] OR "mcp-1"[Title/Abstract] OR (("high-mobility group"[Title/Abstract] AND (b1[Title/Abstract] OR "box 1"[Title/Abstract]))) OR hmgb1[Title/Abstract] OR procalcitonin[Title/Abstract] OR "c-reactive protein"[Title/Abstract] OR lactate[Title/Abstract] OR "lactic acid"[Title/Abstract] OR "tumor necrosis factor alpha"[Title/Abstract] OR "tnf-alpha"[Title/Abstract] OR "soluble triggering receptor expressed on myeloid cells-1"[Title/Abstract] OR strem1[Title/Abstract] OR "soluble urokinase-type plasminogen receptor"[Title/Abstract] OR "soluble urokinase-type plasminogen activator receptor"[Title/Abstract] OR supar[Title/Abstract] OR "soluble upar"[Title/Abstract] OR "angiopoietin 1"[Title/Abstract] OR "angiopoietin 2"[Title/Abstract] OR "ang-1"[Title/Abstract] OR "ang-2"[Title/Abstract])))) OR (("sepsis-related organ failure assessment"[Title/Abstract] OR "sequential organ failure assessment"[Title/Abstract] OR ((sofa[Title/Abstract] AND score*[Title/Abstract])))))) AND (((mortality[Title] OR surviv*[Title])) OR ("length of stay"[Title/Abstract] OR "time spent in icu"[Title/Abstract] OR "time spent in emergency"[Title/Abstract] OR "on machine ventilation"[Title/Abstract] OR ((clinical*[Title/Abstract] AND outcome*[Title/Abstract]))))) AND ((prognos*[Title] OR predict*[Title] OR correlat*[Title] OR accuracy[Title] OR accurate[Title] OR utility[Title] OR useful*[Title] OR value*[Title] OR evidence[Title] OR assess*[Title] OR level*[Title] OR determin*[Title] OR detect*[Title] OR measure*[Title] OR marker*[Title] OR biomarker*[Title] OR evaluat*[Title]))) NOT ((animals[mh] NOT humans[mh]))) AND english[Language]) AND (((inprocess[sb]) OR (publisher[sb] NOT pubstatusnihms NOT pubstatuspmcsd NOT pmcbook)))	54	09:35:21

#8	Add	Search (((((sepsis[Title] OR septic[Title]))) AND (((((biomarker*[Title/Abstract] OR “biological marker*”[Title/Abstract] OR (((interleukin[Title/Abstract] OR il)[Title/Abstract] AND (4[Title/Abstract] OR 6[Title/Abstract] OR 8[Title/Abstract] OR 10[Title/Abstract] OR “receptor antagonist*”[Title/Abstract] OR “blocking agent*”[Title/Abstract]))) OR “advanced glycation endproduct receptor*”[Title/Abstract] OR “advanced glycation end product receptor*”[Title/Abstract] OR “receptor* for advanced glycation endproducts”[Title/Abstract] OR “receptor* for advanced glycation end products”[Title/Abstract] OR rage[Title/Abstract] OR “monocyte chemoattractant protein*”[Title/Abstract] OR “monocyte chemotactic protein*”[Title/Abstract] OR “mcp-1”[Title/Abstract] OR ((“high-mobility group”[Title/Abstract] AND (b1[Title/Abstract] OR “box 1”[Title/Abstract]))) OR hmgb1[Title/Abstract] OR procalcitonin[Title/Abstract] OR “c-reactive protein”[Title/Abstract] OR lactate[Title/Abstract] OR “lactic acid”[Title/Abstract] OR “tumor necrosis factor alpha”[Title/Abstract] OR “tnf-alpha”[Title/Abstract] OR “soluble triggering receptor expressed on myeloid cells-1”[Title/Abstract] OR strem1[Title/Abstract] OR “soluble urokinase-type plasminogen receptor”[Title/Abstract] OR “soluble urokinase-type plasminogen activator receptor”[Title/Abstract] OR supar[Title/Abstract] OR “soluble upar”[Title/Abstract] OR “angiopoietin 1”[Title/Abstract] OR “angiopoietin 2”[Title/Abstract] OR “ang-1”[Title/Abstract] OR “ang-2”[Title/Abstract])))) OR ((“sepsis-related organ failure assessment”[Title/Abstract] OR “sequential organ failure assessment”[Title/Abstract] OR ((sofa[Title/Abstract] AND score*[Title/Abstract])))))) AND (((mortality[Title] OR surviv*[Title])) OR (“length of stay”[Title/Abstract] OR “time spent in icu”[Title/Abstract] OR “time spent in emergency”[Title/Abstract] OR “on machine ventilation”[Title/Abstract] OR ((clinical*[Title/Abstract] AND outcome*[Title/Abstract]))))) AND ((prognos*[Title] OR predict*[Title] OR correlat*[Title] OR accuracy[Title] OR accurate[Title] OR utility[Title] OR useful*[Title] OR value*[Title] OR evidence[Title] OR assess*[Title] OR level*[Title] OR determin*[Title] OR detect*[Title] OR measure*[Title] OR marker*[Title] OR biomarker*[Title] OR evaluat*[Title]))	568	09:33:20
#7	Add	Search ((((biomarker*[Title/Abstract] OR “biological marker*”[Title/Abstract] OR (((interleukin[Title/Abstract] OR il)[Title/Abstract] AND (4[Title/Abstract] OR 6[Title/Abstract] OR 8[Title/Abstract] OR 10[Title/Abstract] OR “receptor antagonist*”[Title/Abstract] OR “blocking agent*”[Title/Abstract]))) OR “advanced glycation endproduct receptor*”[Title/Abstract] OR “advanced glycation end product receptor*”[Title/Abstract] OR “receptor* for advanced glycation endproducts”[Title/Abstract] OR “receptor* for advanced glycation end products”[Title/Abstract] OR rage[Title/Abstract] OR “monocyte chemoattractant protein*”[Title/Abstract] OR “monocyte chemotactic protein*”[Title/Abstract] OR “mcp-1”[Title/Abstract] OR ((“high-mobility group”[Title/Abstract] AND (b1[Title/Abstract] OR “box 1”[Title/Abstract]))) OR hmgb1[Title/Abstract] OR procalcitonin[Title/Abstract] OR “c-reactive protein”[Title/Abstract] OR lactate[Title/Abstract] OR “lactic acid”[Title/Abstract] OR “tumor necrosis factor alpha”[Title/Abstract] OR “tnf-alpha”[Title/Abstract] OR “soluble triggering receptor expressed on myeloid cells-1”[Title/Abstract] OR strem1[Title/Abstract] OR “soluble urokinase-type plasminogen receptor”[Title/Abstract] OR “soluble urokinase-type plasminogen activator receptor”[Title/Abstract] OR supar[Title/Abstract] OR “soluble upar”[Title/Abstract] OR “angiopoietin 1”[Title/Abstract] OR “angiopoietin 2”[Title/Abstract] OR “ang-1”[Title/Abstract] OR “ang-2”[Title/Abstract])))) OR ((“sepsis-related organ failure assessment”[Title/Abstract] OR “sequential organ failure assessment”[Title/Abstract] OR ((sofa[Title/Abstract] AND score*[Title/Abstract]))))	497763	09:32:47
#6	Add	Search (prognos*[Title] OR predict*[Title] OR correlat*[Title] OR accuracy[Title] OR accurate[Title] OR utility[Title] OR useful*[Title] OR value*[Title] OR evidence[Title] OR assess*[Title] OR level*[Title] OR determin*[Title] OR detect*[Title] OR measure*[Title] OR marker*[Title] OR biomarker*[Title] OR evaluat*[Title])	2858431	09:10:30
#5	Add	Search ((mortality[Title] OR surviv*[Title])) OR (“length of stay”[Title/Abstract] OR “time spent in icu”[Title/Abstract] OR “time spent in emergency”[Title/Abstract] OR “on machine ventilation”[Title/Abstract] OR ((clinical*[Title/Abstract] AND outcome*[Title/Abstract])))	780175	09:08:10

#4	Add	Search **((biomarker*[Title/Abstract] OR “biological marker*”[Title/Abstract] OR (((interleukin[Title/Abstract] OR il)[Title/Abstract] AND (4[Title/Abstract] OR 6[Title/Abstract] OR 8[Title/Abstract] OR 10[Title/Abstract] OR “receptor antagonist*”[Title/Abstract] OR “blocking agent*”[Title/Abstract]))) OR “advanced glycation endproduct receptor*”[Title/Abstract] OR “advanced glycation end product receptor*”[Title/Abstract] OR “receptor* for advanced glycation endproducts”[Title/Abstract] OR “receptor* for advanced glycation end products”[Title/Abstract] OR rage[Title/Abstract] OR “monocyte chemoattractant protein*”[Title/Abstract] OR “monocyte chemotactic protein*”[Title/Abstract] OR “mcp-1”[Title/Abstract] OR ((“high-mobility group”[Title/Abstract] AND (b1[Title/Abstract] OR “box 1”[Title/Abstract]))) OR hmgb1[Title/Abstract] OR procalcitonin[Title/Abstract] OR “c-reactive protein”[Title/Abstract] OR lactate[Title/Abstract] OR “lactic acid”[Title/Abstract] OR “tumor necrosis factor alpha”[Title/Abstract] OR “tnf-alpha”[Title/Abstract] OR “soluble triggering receptor expressed on myeloid cells-1”[Title/Abstract] OR strem1[Title/Abstract] OR “soluble urokinase-type plasminogen receptor”[Title/Abstract] OR “soluble urokinase-type plasminogen activator receptor”[Title/Abstract] OR supar[Title/Abstract] OR “soluble upar”[Title/Abstract] OR “angiopoietin 1”[Title/Abstract] OR “angiopoietin 2”[Title/Abstract] OR “ang-1”[Title/Abstract] OR “ang-2”[Title/Abstract]))**	495700	09:04:50
#3	Add	Search **(“sepsis-related organ failure assessment”[Title/Abstract] OR “sequential organ failure assessment”[Title/Abstract] OR ((sofa[Title/Abstract] AND score*[Title/Abstract])))**	2843	09:02:02
#1	Add	Search **(sepsis[Title] OR septic[Title])**	40873	08:59:31

You are here: NCBI > Literature > PubMed
Support Center

GETTING STARTED
NCBI Education
NCBI Help Manual
NCBI Handbook
Training & Tutorials
Submit Data

RESOURCES
Chemicals & Bioassays
Data & Software
DNA & RNA
Domains & Structures
Genes & Expression
Genetics & Medicine
Genomes & Maps
Homology
Literature
Proteins
Sequence Analysis
Taxonomy
Variation

POPULAR
PubMed
Bookshelf
PubMed Central
PubMed Health
BLAST
Nucleotide
Genome
SNP
Gene
Protein
PubChem

FEATURED
Genetic Testing Registry
PubMed Health
GenBank
Reference Sequences
Gene Expression Omnibus
Map Viewer
Human Genome
Mouse Genome
Influenza Virus
Primer-BLAST
Sequence Read Archive

NCBI INFORMATION
About NCBI
Research at NCBI
NCBI News & Blog
NCBI FTP Site
NCBI on Facebook
NCBI on Twitter
NCBI on YouTube

National Center for Biotechnology Information, U.S. National Library of Medicine
8600 Rockville Pike, Bethesda MD, 20894 USA
Policies and Guidelines | **Contact**
