# Supplementary material for: Prediction of mortality in adult patients with sepsis using six biomarkers: a systematic review and meta-analysis
Source: Ann Intensive Care. 2019 Nov 8;9:125. doi: 10.1186/s13613-019-0600-1 (PMC6841861; doi:10.1186/s13613-019-0600-1)
Supplement: Supplementary file 2 — Additional file 2. List of excluded articles, with reasons. [file 13613_2019_600_MOESM2_ESM.pdf]

Additional file 2 - List of excluded articles, with reasons:

Reviews and Systematic reviews

1. Adams CA, (2011) Sepsis Biomarkers in Polytrauma Patients. Critical Care Clinics 27: 345-354
2. Backes Y, Van Der Sluijs KF, Mackie DP, Tacke F, Koch A, Tenhunen JJ, Schultz MJ, (2012) Usefulness of suPAR as a biological marker in patients with systemic inflammation or infection: A systematic review. Intensive Care Medicine 38: 1418-1428
3. Christ-Crain M, Müller B, (2007) Biomarkers in respiratory tract infections: Diagnostic guides to antibiotic prescription, prognostic markers and mediators. European Respiratory Journal 30: 556-573
4. Ciriello V, Gudipati S, Stavrou PZ, Kanakaris NK, Bellamy MC, Giannoudis PV, (2013) Biomarkers predicting sepsis in polytrauma patients: Current evidence. Injury 44: 1680-1692
5. Esposito S, De Simone G, Boccia G, De Caro F, Pagliano P, (2017) Sepsis and septic shock: New definitions, new diagnostic and therapeutic approaches. Journal of Global Antimicrobial Resistance 10: 204-212
6. Eugen-Olsen J, Giamarellos-Bourboulis EJ, (2015) SuPAR: The unspecific marker for disease presence, severity and prognosis. International Journal of Antimicrobial Agents 46: S33-S34
7. Faix JD, (2013) Biomarkers of sepsis. Critical Reviews in Clinical Laboratory Sciences 50: 23-36
8. Faix JD, (2011) Established and novel biomarkers of sepsis. Biomarkers in Medicine 5: 117-130
9. Fan SL, Miller NS, Lee J, Remick DG, (2016) Diagnosing sepsis – The role of laboratory medicine. Clinica Chimica Acta 460: 203-210
10. Giannakopoulos K, Hoffmann U, Ansari U, Bertsch T, Borggrefe M, Akin I, Behnes M, (2017) The use of biomarkers in sepsis: A systematic review. Current Pharmaceutical Biotechnology 18: 499-507
11. Iskander KN, Osuchowski MF, Stearns-Kurosawa DJ, Kurosawa S, Stepien D, Valentine C, Remick DG, (2013) Sepsis: Multiple abnormalities, heterogeneous responses, and evolving understanding. Physiological Reviews 93: 1247-1288
12. Jacobi J, (2002) Sepsis: A frequent, life-threatening syndrome. Pharmacotherapy 22: 169S-181S
13. Lee WL, Liles WC, (2011) Endothelial activation, dysfunction and permeability during severe infections. Current Opinion in Hematology 18: 191-196
14. Lichtenstern C, Brenner T, Bardenheuer HJ, Weigand MA, (2012) Predictors of survival in sepsis: what is the best inflammatory marker to measure? Current opinion in infectious diseases 25: 328-336
15. Nylén ES, Seam N, Khosla R, (2006) Endocrine markers of severity and prognosis in critical illness. Critical Care Clinics 22: 161-179
16. Reinhart K, Bauer M, Riedemann NC, Hartog CS, (2012) New approaches to sepsis: Molecular diagnostics and biomarkers. Clinical Microbiology Reviews 25: 609-634
17. Reinhart K, Meisner M, Brunkhorst FM, (2006) Markers for Sepsis Diagnosis: What is Useful? Critical Care Clinics 22: 503-519
18. Schuetz P, Christ-Crain M, Müller B, (2009) Procalcitonin and other biomarkers to improve assessment and antibiotic stewardship in infections - Hope for hype? Swiss Medical Weekly 139: 318-326
19. Su L, Liu D, Chai W, Liu D, Long Y, (2016) Role of sTREM-1 in predicting mortality of infection: A systematic review and meta-analysis. BMJ Open 6
20. Tziolos N, Kotanidou A, Orfanos SE, (2015) Biomarkers in infection and sepsis: Can they really indicate final outcome? International Journal of Antimicrobial Agents 46: S29-S32
21. Vera S, Martinez R, Gormaz JG, Gajardo A, Galleguillos F, Rodrigo R, (2015) Novel relationships between oxidative stress and angiogenesis-related factors in sepsis: New biomarkers and therapies. Annals of Medicine 47: 289-300
22. Wu Y, Wang F, Fan X, Bao R, Bo L, Li J, Deng X, (2012) Accuracy of plasma sTREM-1 for sepsis diagnosis in systemic inflammatory patients: a systematic review and meta-analysis. Critical care (London, England) 16: R229
23. Xing K, Murthy S, Liles WC, Singh JM, (2012) Clinical utility of biomarkers of endothelial activation in sepsis--a systematic review. Critical care (London, England) 16: R7

Commentaries/Posters/Abstracts/Editorials/Letters

24. David S, van Meurs M, Kümpers P, (2010) Does low angiopoietin-1 predict adverse outcome in sepsis? Critical Care 14
25. de la Torre-Prados MV, García-de la Torre A, Puerto-Morlán A, Cámara-Sola E, Nuevo-Ortega P, Tsvetanova Spasova T, Férnandez-Porcel A, Rueda-Molina C, García-Alcántara A, (2015) Biomarkers mesuared within 24 hours from the onset of septic shock and 28-day mortality. Intensive Care Medicine Experimental 3

26. Douglas J, Fisher J, Boyd J, Russell J, Walley K, (2014) Elevated plasma ANG-2 levels are associated with fluid overload, organ dysfunction and mortality in human septic shock. American journal of respiratory and critical care medicine DOI
27. Eugen-Olsen J, (2011) suPAR - a future risk marker in bacteremia. Journal of Internal Medicine 270: 29-31
28. Garg G, Gogia A, Kakar A, (2015) Soluble urokinase plasminogen activator receptor: A new biomarker. Current Medicine Research and Practice 5: 50
29. Hahn WO, Mikacenic C, Price BL, Harju-Baker S, Katz R, Himmelfarb J, Wurfel MM, Liles WC, (2016) Host derived biomarkers of inflammation, apoptosis, and endothelial activation are associated with clinical outcomes in patients with bacteremia and sepsis regardless of microbial etiology. Virulence 7: 387-394
30. Nielsen VG, (2015) Old mineshaft, new canary: Can circulating osteopontin concentrations predict septic shock? Minerva Anestesiologica 81: 116-118
31. Sherif HM, Farghal A, Al Sisi A, Al Maraghy S, (2015) Urinary strem-1 is an early outcome predictor for sepsis and sepsis-induced acute kidney injury. Intensive Care Medicine Experimental 3
32. Siner JM, (2013) A tale of two ligands: Angiopoietins, the endothelium, and outcomes. Critical Care 17
33. Zhang RY, Zhang H, Huang J, Qu HP, Tang YQ, (2014) Angiogenic factors in sepsis: Are we ready for the new therapeutic era? Critical Care 18

Data from interventional studies

34. Ingels C, Derese I, Wouters PJ, Van den Berghe G, Vanhorebeek I, (2015) Soluble RAGE and the RAGE ligands HMGB1 and S100A12 in critical illness: impact of glycemic control with insulin and relation with clinical outcome. Shock (Augusta, Ga) 43: 109-116
35. Fisher J, Douglas JJ, Linder A, Boyd JH, Walley KR, Russell JA, (2016) Elevated Plasma Angiopoietin-2 Levels Are Associated With Fluid Overload, Organ Dysfunction, and Mortality in Human Septic Shock. Critical care medicine 44: 2018-2027
36. Laudanski K, Chalupka A, Nielson V, Novack V, Talmor D, (2012) Pilot study of the continuous infusion of ketamine on the cytokine levels in patient with sirs/sepsis. Critical care medicine DOI 10.1097/01.ccm.0000425605.04623.4b

No biomarker of interest

37. Dalli J, Colas RA, Quintana C, Barragan-Bradford D, Hurwitz S, Levy BD, Choi AM, Serhan CN, Baron RM, (2017) Human Sepsis Eicosanoid and Proresolving Lipid Mediator Temporal Profiles: Correlations With Survival and Clinical Outcomes. Critical care medicine 45: 58-68
38. Karampela I, Kandri E, Antonakos G, Vogiatzakis E, Christodoulatos GS, Nikolaidou A, Dimopoulos G, Armaganidis A, Dalamaga M, (2017) Kinetics of circulating fetuin-A may predict mortality independently from adiponectin, high molecular weight adiponectin and prognostic factors in critically ill patients with sepsis: A prospective study. Journal of critical care 41: 78-85
39. Koch A, Weiskirchen R, Bruensing J, Dückers H, Buendgens L, Kunze J, Matthes M, Luedde T, Trautwein C, Tacke F, (2013) Regulation and prognostic relevance of symmetric dimethylarginine serum concentrations in critical illness and sepsis. Mediators of Inflammation 2013
40. Koch A, Weiskirchen R, Kunze J, Duckers H, Bruensing J, Buendgens L, Matthes M, Luedde T, Trautwein C, Tacke F, (2013) Elevated asymmetric dimethylarginine levels predict short- and long-term mortality risk in critically ill patients. Journal of critical care 28: 947-953
41. Koch A, Weiskirchen R, Ludwig S, Buendgens L, Bruensing J, Yagmur E, Baeck C, Herbers U, Trautwein C, Tacke F, (2017) Relevance of serum sclerostin concentrations in critically ill patients. Journal of critical care 37: 38-44
42. Lebherz C, Schlieper G, Mollmann J, Kahles F, Schwarz M, Brunsing J, Dimkovic N, Koch A, Trautwein C, Floge J, Marx N, Tacke F, Lehrke M, (2017) GLP-1 Levels Predict Mortality in Patients with Critical Illness as Well as End-Stage Renal Disease. The American journal of medicine 130: 833-841.e833
43. Nagai M, Hirayama K, Ebihara I, Higuchi T, Imaizumi M, Maruyama H, Miyamoto Y, Kakita T, Ogawa Y, Fujita S, Shimohata H, Kobayashi M, (2011) Serum TNF-Related and Weak Inducer of Apoptosis Levels in Septic Shock Patients. Therapeutic Apheresis and Dialysis 15: 342-348
44. Nguyen HB, Loomba M, Yang JJ, Jacobsen G, Shah K, Otero RM, Suarez A, Parekh H, Jaehne A, Rivers EP, (2010) Early lactate clearance is associated with biomarkers of inflammation, coagulation, apoptosis, organ dysfunction and mortality in severe sepsis and septic shock. Journal of inflammation (London, England) 7: 6

45. Roderburg C, Benz F, Cardenas DV, Lutz M, Hippe HJ, Luedde T, Trautwein C, Frey N, Koch A, Tacke F, Luedde M, (2015) Persistently elevated osteopontin serum levels predict mortality in critically ill patients. Critical Care 19
46. Yagmur E, Koch A, Haumann M, Kramann R, Trautwein C, Tacke F, (2012) Hyaluronan serum concentrations are elevated in critically ill patients and associated with disease severity. Clinical biochemistry 45: 82-87
47. Yang K-Y, Liu K-T, Chen Y-C, Chen C-S, Lee Y-C, Perng R-P, Feng J-Y, (2011) Plasma soluble vascular endothelial growth factor receptor-1 levels predict outcomes of pneumonia-related septic shock patients: a prospective observational study. Critical care (London, England) 15: R11

No sepsis patients/Mixed cohort

48. Gaïni S, Koldkjær OG, Møller HJ, Pedersen C, Pedersen SS, (2007) A comparison of high-mobility group-box 1 protein, lipopolysaccharide-binding protein and procalcitonin in severe community-acquired infections and bacteraemia: A prospective study. Critical Care 11
49. Gaïni S, Pedersen SS, Koldkjær OG, Pedersen C, Moestrup SK, Møller HJ, (2008) New immunological serum markers in bacteraemia: Anti-inflammatory soluble CD163, but not proinflammatory high mobility group-box 1 protein, is related to prognosis. Clinical and Experimental Immunology 151: 423-431
50. Giamarellos-Bourboulis EJ, Kanellakopoulou K, Pelekanou A, Tsaganos T, Kotzampassi K, (2008) Kinetics of angiopoietin-2 in serum of multi-trauma patients: correlation with patient severity. Cytokine 44: 310-313
51. Huttunen R, Syrjanen J, Vuento R, Hurme M, Huhtala H, Laine J, Pessi T, Aittoniemi J, (2011) Plasma level of soluble urokinase-type plasminogen activator receptor as a predictor of disease severity and case fatality in patients with bacteraemia: a prospective cohort study. J Intern Med 270: 32-40
52. Koch A, Voigt S, Kruschinski C, Sanson E, Duckers H, Horn A, Yagmur E, Zimmermann H, Trautwein C, Tacke F, (2011) Circulating soluble urokinase plasminogen activator receptor is stably elevated during the first week of treatment in the intensive care unit and predicts mortality in critically ill patients. Critical care (London, England) 15: R63
53. Kofoed K, Eugen-Olsen J, Petersen J, Larsen K, Andersen O, (2008) Predicting mortality in patients with systemic inflammatory response syndrome: an evaluation of two prognostic models, two soluble receptors, and a macrophage migration inhibitory factor. Eur J Clin Microbiol Infect Dis 27: 375-383
54. Kümpers P, Hafer C, David S, Hecker H, Lukasz A, Fliser D, Haller H, Kielstein JT, Faulhaber-Walter R, (2010) Angiopoietin-2 in patients requiring renal replacement therapy in the ICU: Relation to acute kidney injury, multiple organ dysfunction syndrome and outcome. Intensive Care Medicine 36: 462-470
55. Kümpers P, Lukasz A, David S, Horn R, Hafer C, Faulhaber-Walter R, Fliser D, Haller H, Kielstein JT, (2008) Excess circulating angiopoietin-2 is a strong predictor of mortality in critically ill medical patients. Critical Care 12
56. Lin MT, Wei YF, Ku SC, Lin CA, Ho CC, Yu CJ, (2010) Serum soluble triggering receptor expressed on myeloid cells-1 in acute respiratory distress syndrome: A prospective observational cohort study. Journal of the Formosan Medical Association 109: 800-809
57. Molkanen T, Ruotsalainen E, Thorball CW, Jarvinen A, (2011) Elevated soluble urokinase plasminogen activator receptor (suPAR) predicts mortality in Staphylococcus aureus bacteremia. European journal of clinical microbiology & infectious diseases : official publication of the European Society of Clinical Microbiology 30: 1417-1424
58. Moller HJ, Moestrup SK, Weis N, Wejse C, Nielsen H, Pedersen SS, Attermann J, Nexo E, Kronborg G, (2006) Macrophage serum markers in pneumococcal bacteremia: Prediction of survival by soluble CD163. Critical care medicine 34: 2561-2566
59. Raggam RB, Wagner J, Prüller F, Grisold A, Leitner E, Zollner-Schwetz I, Valentin T, Krause R, Hoenigl M, (2014) Soluble urokinase plasminogen activator receptor predicts mortality in patients with systemic inflammatory response syndrome. Journal of Internal Medicine 276: 651-658
60. Suda K, Kitagawa Y, Ozawa S, Saikawa Y, Ueda M, Abraham E, Kitajima M, Ishizaka A, (2006) Serum concentrations of high-mobility group box chromosomal protein 1 before and after exposure to the surgical stress of thoracic esophagectomy: A predictor of clinical course after surgery? Diseases of the Esophagus 19: 5-9
61. van der Heijden M, van Nieuw Amerongen GP, Koolwijk P, van Hinsbergh VW, Groeneveld AB, (2008) Angiopoietin-2, permeability oedema, occurrence and severity of ALI/ARDS in septic and non-septic critically ill patients. Thorax 63: 903-909
62. Wittenhagen P, Kronborg G, Weis N, Nielsen H, Obel N, Pedersen SS, Eugen-Olsen J, (2004) The plasma level of soluble urokinase receptor is elevated in patients with Streptococcus pneumoniae

bacteraemia and predicts mortality. Clinical microbiology and infection : the official publication of the European Society of Clinical Microbiology and Infectious Diseases 10: 409-415
63. Wu XL, Long D, Yu L, Yang JH, Zhang YC, Geng F, (2013) Urokinase-type plasminogen activator receptor as a predictor of poor outcome in patients with systemic infl ammatory response syndrome. World Journal of Emergency Medicine 4: 190-195

No prognostic information

64. Chen Q, Ye L, Jin Y, Zhang N, Lou T, Qiu Z, Jin Y, Cheng B, Fang X, (2012) Circulating nucleosomes as a predictor of sepsis and organ dysfunction in critically ill patients. International journal of infectious diseases : IJID : official publication of the International Society for Infectious Diseases 16: e558-564
65. Wiewel MA, de Stoppelaar SF, van Vught LA, Frencken JF, Hoogendijk AJ, Klein Klouwenberg PMC, Horn J, Bonten MJ, Zwinderman AH, Cremer OL, Schultz MJ, van der Poll T, (2016) Chronic antiplatelet therapy is not associated with alterations in the presentation, outcome, or host response biomarkers during sepsis: a propensity-matched analysis. Intensive Care Medicine 42: 352-360
66. Wada T, Jesmin S, Gando S, Yanagida Y, Mizugaki A, Sultana SN, Zaedi S, Yokota H, (2013) The role of angiogenic factors and their soluble receptors in acute lung injury (ALI)/ acute respiratory distress syndrome (ARDS) associated with critical illness. Journal of Inflammation (United Kingdom) 10
67. Mikacenic C, Price BL, Harju-Baker S, O'Mahony DS, Robinson-Cohen C, Radella F, Hahn WO, Katz R, Christiani DC, Himmelfarb J, Liles WC, Wurfel MM, (2017) A Two-Biomarker Model Predicts Mortality in the Critically Ill with Sepsis. American journal of respiratory and critical care medicine 196: 1004-1011
68. Orfanos SE, Kotanidou A, Glynos C, Athanasiou C, Tsigkos S, Dimopoulou I, Sotiropoulou C, Zakynthinos S, Armaganidis A, Papapetropoulos A, Roussos C, (2007) Angiopoietin-2 is increased in severe sepsis: correlation with inflammatory mediators. Crit Care Med 35: 199-206

Overlapping cohorts

69. Su L, Han B, Liu C, Liang L, Jiang Z, Deng J, Yan P, Jia Y, Feng D, Xie L, (2012) Value of soluble TREM-1, procalcitonin, and C-reactive protein serum levels as biomarkers for detecting bacteremia among sepsis patients with new fever in intensive care units: a prospective cohort study. BMC Infectious Diseases 12
70. Su LX, Meng K, Zhang X, Wang HJ, Yan P, Jia YH, Feng D, Xie LX, (2012) Diagnosing ventilator-associated pneumonia in critically ill patients with sepsis. American journal of critical care : an official publication, American Association of Critical-Care Nurses 21: e110-119
